# Community as Medicine: A Qualitative Study of How Group Health Coaching and Social Connection Improve Mental Well-Being in Older Adults

**DOI:** 10.3390/healthcare14040510

**Published:** 2026-02-17

**Authors:** Sally C. Duplantier, Michaela G. Hayes, Noriah Sanchez-Zaragoza, Angelina I. Londoño, Erykah Hamilton, Elizabeth A. Markle, Benjamin Emmert-Aronson

**Affiliations:** 1 Fairbanks School of Public Health, Indiana University, Indianapolis, IN 46202, USA; 2 Open Source Wellness, 6921 Snowdon Avenue, El Cerrito, CA 94530, USA; michaela@opensourcewellness.org (M.G.H.); angelina@ixikwellness.com (A.I.L.); liz@opensourcewellness.org (E.A.M.); ben@opensourcewellness.org (B.E.-A.); 3 The USC Leonard School of Gerontology, University of Southern California, Los Angeles, CA 90089, USA; nszarago@usc.edu

**Keywords:** mental well-being, mental health, Community As Medicine^®^, social isolation, older adults, internalized ageism, social learning, qualitative research

## Abstract

Background/Objectives. Older adults in under-resourced communities experience high levels of social isolation, chronic illness, and reduced access to healthcare, which can undermine mental well-being. Open Source Wellness’s Community As Medicine^®^ (CAM) program is an evidence-based, community-delivered, clinically integrated program that combines trauma-informed, culturally-relevant, experiential group health coaching with social connection to improve mental and physical well-being. This qualitative study, conducted in early 2025, examined how participation in CAM supports mental well-being among older adults (age 65+) in under-resourced communities who are managing chronic physical and mental health challenges. Methods. Semi-structured interviews were conducted with participants who completed CAM. Transcripts were analyzed using reflexive thematic analysis to explore relational and experiential processes associated with improved well-being. Findings. Participants entered CAM with internalized ageist beliefs and low expectations for personal change. As they engaged in new behaviors, experienced successes, and observed similar progress among peers, they gained motivation, confidence, and a sense of control. Four interconnected themes appear to explain the mechanisms through which CAM supports mental well-being: (1) belonging and inclusion through trust and safety; (2) personal accountability through relational accountability; (3) self-efficacy through social learning and reciprocal support; and (4) agency through positive actions. Conclusions. Findings suggest that CAM supports mental well-being by creating conditions that help older adults overcome internalized ageism and feel connected, capable, and in control of their lives. These results identify actionable strategies that community organizations and health systems can adapt to support mental well-being for older adults in under-resourced communities.

## 1. Introduction

Social isolation and loneliness are increasingly recognized as urgent public health challenges, particularly for older adults [1,2,3]. While social isolation and loneliness can affect people of all ages, older adults are especially vulnerable because of declining mobility, shrinking social networks due to loss of family members and friends, and changes in social roles after retirement that make it harder to stay connected [4]. Social isolation (the objective lack of social contact) and loneliness (the subjective feeling of disconnection) are distinct but often co-occur [5]. Both conditions undermine the physical, mental, and cognitive health of older adults [6,7,8], contributing to diminished quality of life [9,10] and increased morbidity and mortality [6,11].

However, social isolation and loneliness tell only part of the story. A growing body of research indicates that older adults’ emotional well-being is shaped by more profound psychosocial experiences including decreased sense of relevance and internalized ageism—e.g., the internalization of the prevalent societal belief that aging makes one less capable or less valuable [12,13,14]. These experiences and beliefs reduce self-efficacy and increase psychological distress, and can even worsen physical health [12,13,14]. Conversely, mental well-being improves when older adults experience belonging, purpose, and opportunities to support others [15,16,17]. These relational dynamics often emerge through peer-based interactions, where observing, encouraging, and learning from others may reinforce self-efficacy and help shape health-related beliefs and behaviors [15,16,17]. What matters is creating environments that support older adults in overcoming internalized ageism and where they feel seen, welcomed, valued, and capable [14,17,18]. Further, this deeper dimension of social connection–belonging and mattering–has been largely overlooked in interventions that focus solely on increasing social contact [19,20].

Older adults living in under-resourced communities often face additional, compounding challenges that undermine mental well-being. Many reside in neighborhoods shaped by long-standing economic disadvantage, systemic racism, and migration-related stress [21,22,23]. These community conditions, widely recognized as social determinants of health (SDOH), contribute to higher burdens of chronic disease and depressive symptoms among low-income older adults [3,21,24]. National data show that even when older adults meet clinical criteria for mood or anxiety disorders, many receive no mental health treatment; lower-income older adults are particularly likely to go untreated [25]. Homebound, low-income older adults are especially vulnerable; among one sample, nearly half reported no mental health service use in the previous year [26]. Additionally, older adults’ access to mental health care is often limited by structural barriers such as cost, lack of insurance coverage or being under-insured, provider shortages, and mobility constraints—challenges that are particularly pronounced in under-resourced settings [27,28]. These factors highlight the importance of considering the broader social and environmental context in which mental well-being is shaped. Collectively, these conditions create a critical gap in accessible mental health support for older adults who reside in under-resourced communities. This underscores the need for accessible community-based approaches delivered by less costly paraprofessionals, such as health coaches and community health workers, that complement clinical care and address complex health and social needs not typically met in traditional healthcare settings.

This qualitative study examines how Community as Medicine^®^ (CAM)—an evidence-based, community-delivered, clinically integrated program developed by Open Source Wellness—promotes mental well-being in older adults living in under-resourced communities. Specifically, CAM combines group health coaching with structured social connection, playful exercise adaptable to multiple ability levels, mindfulness-based stress reduction, and nutrition education to improve mental and physical health and well-being [29]. Although the CAM program serves adults of all ages in an intergenerational format, this study examines the experiences of older adults (age 65+) participating in the CAM program. Prior evaluations of the CAM program have focused on participants across age categories and have revealed improvements in mental well-being associated with reductions in depression, anxiety, and isolation; improvements in healthy eating (measured by increased consumption of fruits and vegetables); and increased levels of physical activity [30]. What remains unknown is how these improvements occur. Accordingly, this study asks: How does participation in the CAM program support mental well-being among older adults living in under-resourced communities? Rather than attributing benefits solely to participation or social contact, the analysis examines the relational and experiential mechanisms through which well-being is supported in a structured group health coaching environment.

### The CAM Model

This study focused on a sample of older adult participants in Open Source Wellness’s (OSW’s) Community As Medicine^®^ (CAM) program in Alameda County, California. The OSW CAM program (a.k.a. “CAM program”) is an evidence-based lifestyle medicine [31] intervention that seeks to improve physical, mental, and social well-being in partnership with clinical healthcare and community-based organizations. Consistent with ecological theories of human development [32] and the Social Ecological Model widely used in health promotion [33], OSW’s CAM program is designed to intervene across multiple levels of influence—individual, interpersonal, and community—creating conditions that support positive behavior change and improved mental well-being.

The CAM program in Alameda County is designed to serve residents of under-resourced communities who have a variety of chronic mental and/or physical conditions that are directly associated with various underlying SDOH. It is delivered in partnership with Recipe4Health (R4H), an initiative of Alameda County Health, the county’s public health department. Participants are patients of local federally qualified health center (FQHC) clinics, which largely, but not exclusively, serve enrollees in Medi-Cal (California’s Medicaid program) and the uninsured population.

The standard CAM direct service program consists of weekly 1–2 h group health coaching sessions spanning a 12-week period. Providers at partner clinics refer patients who meet program eligibility requirements to the CAM program. Essentially, the patient’s clinical care provider gives them a “prescription” to participate in the CAM program. Eligibility requirements include having a diagnosis of a chronic physical condition such as diabetes, pre-diabetes, and/or hypertension, and/or chronic depression. Via the CAM program, participants (*n* = 12–24 per group) engage in four “pillars” of lifestyle medicine: physical activity (move), healthy nutrition (nourish), social connection (connect), and stress reduction (be). Groups are intergenerational; participants’ ages range from 18 to 80+. The health coach supports participants in developing self-directed health-related goals and associated actions towards improved health and well-being. These goals are reviewed on an ongoing basis during the participant’s engagement in the CAM program. In addition to the health coach, the CAM groups may include a peer leader who assists the health coach. Peer leaders are former participants who have “graduated” from CAM, i.e., who have completed a 12-session “dose” of the CAM program, and applied and been accepted to the peer leader program. Groups are available in English, Spanish, and Cantonese; in-person and virtually; and during the weekdays, evenings, and Saturdays.

During each clinically integrated CAM group health coaching session, each participant leaves the group for a short period of time during which they meet 1:1 with a primary care provider for a brief medical visit to monitor their health and measure changes in key metrics (e.g., blood pressure). Many clinical partners choose to incorporate the CAM group into a group medical visit (GMV) to support patient care, provider experience, and financial sustainability [34].

**Figure 1:**
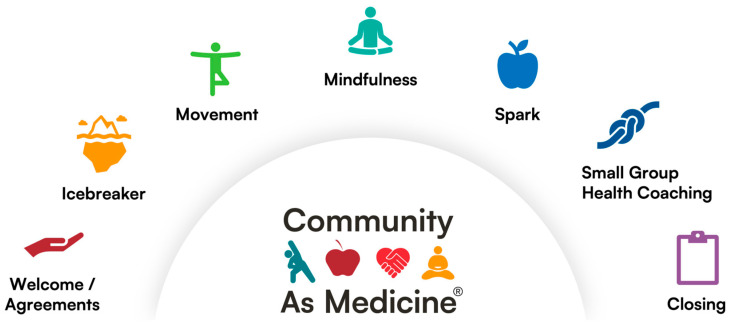
The arc of a Community As Medicine^®^ program session.

Each CAM session follows a specific sequence of activities: the “arc” of the CAM group session (Figure 1). In the first half of the session, the full group of participants (*n* = 12–24) meets together for a short welcome and icebreaker. This is followed by 15–20 min of physical activity designed to accommodate the variety of mobility levels of participants; a short period of guided mindfulness meditation; a 10 min interactive lesson (a “spark”) on a health topic related to the four pillars, such as nutrition, which is led by a health coach; and preparation of a healthy snack. Following this, the group breaks into small groups (e.g., 4–6 participants) for a group health coaching session, which spans 30–45 min. Each small group is facilitated by a health coach. During the small group sessions, the participants discuss their progress on their health goals and share information and suggestions to support fellow participants in achieving their goals. All participants come back together to share key learnings from their small group discussions and share affirmations, declarations, gratitude, and/or victories. During the week the health coach typically checks in on each participant via a text message.

## 2. Methods

### 2.1. Design

This study employed a secondary qualitative analysis of existing interview transcripts. Secondary analysis of qualitative data is a well-established approach that extends the value of participants’ contributions, enables new insights to be revealed, and addresses research questions beyond the scope of the initial project [35,36,37]. Secondary data analysis is appropriate for this study because it allows us to analyze data originally collected for a program evaluation through a different lens, exploring how participation in a clinically integrated, group-based health coaching program shaped the mental well-being of older adults living in under-resourced communities.

### 2.2. Data Source and Original Study

The interview transcripts were originally collected between February 7 and 26 February 2025, as part of a grant-funded program evaluation of the CAM program in Alameda County, CA. The original evaluation examined how well the CAM program addressed the needs and preferences of older adults (age 65+), while also identifying areas where adjustments to program design might be warranted to address any identified needs and preferences and gaps in support. A total of ten (10) participants met the criteria for the secondary study that is the focus of this paper. All 10 had attended at least 10 CAM sessions at the time of the interview, although the timeframe in which the participants engaged in the CAM program varied. Participants provided informed consent for the original program evaluation activities conducted by Open Source Wellness.

### 2.3. Interview Process: Original Study

Each participant completed an individual, one-on-one in-depth semi-structured interview which lasted approximately one hour and that was audio-recorded with permission. The interview guide was developed by the lead researcher (S.C.D.), who holds formal training in qualitative research and extensive experience in qualitative instrument development. Questions were intentionally open-ended and non-leading to elicit participants’ perspectives and lived experiences. The draft guide was reviewed by the research team to ensure clarity, relevance, and alignment with the study aims. All interviews were conducted individually rather than in a group or focus group format. Interviews were conducted by a bilingual interviewer (A.I.L.) in English or Spanish, depending on participants’ preferred language. Interviews were transcribed verbatim into their local language using Rev.com’s (version 7.2.X) automated transcription service, which was accessed on the same date that the interview was conducted. All transcripts were reviewed and edited by the interviewer to ensure accuracy prior to analysis.

### 2.4. Present Study Focus

The present study re-analyzed de-identified transcripts from the prior program evaluation to explore how the CAM program supports the mental well-being of older adults living in under-resourced communities. While the original evaluation focused on participants’ needs, preferences, and experiences of the CAM program, this secondary analysis shifted attention toward understanding how participation in the CAM program influenced mental well-being, as described in the voices of older adult participants themselves. Secondary analysis allowed us to revisit existing data with new analytic priorities and to generate deeper insight into the experiential and relational mechanisms through which participation in the CAM program improves mental well-being.

### 2.5. Present Study Translation Process

For the secondary data analysis, Spanish-language interviews were manually transcribed into English by the bilingual interviewer [A.I.L.] to ensure both linguistic accuracy and preservation of meaning (since lead authors S.C.D. and M.G.H. do not speak Spanish). The interviewer has over 23 years of professional experience in translation and interpretation. When literal translation did not fully capture the participant’s intent, the interviewer added interpretive notations to convey cultural or contextual meaning. This translation process reflects established best practices in qualitative research, where careful transcription, translation, and annotation are essential to preserve participants’ voices and ensure analytic validity [38,39]. For the interviews conducted in English, we used the de-identified Rev.com AI-generated transcripts which we checked and lightly corrected to ensure accuracy prior to analysis.

### 2.6. Participants and Eligibility Criteria

For this secondary analysis, only transcripts of participants who met specific eligibility criteria were included. Participants were eligible if they had attended at least ten (10) sessions of the CAM program and were age 65 and older. Ten (10) participants met these criteria; all were included in the present study.

### 2.7. Ethical Considerations

To conduct this analysis, only fully de-identified transcripts were accessed; no direct identifiers or re-identification codes were available to the research team. The project was reviewed by the Indiana University Institutional Review Board (IRB) on 29 September 2025, and determined not to constitute human subjects research because it involved analysis of de-identified data only. As such, formal IRB review and approval were not required.

### 2.8. Data Analysis

Our analysis examined patterns of similarity and divergence in how older adult participants described their experiences with the CAM program and its impact on their mental well-being. Because this was a secondary analysis, we began with 122 pages of de-identified interview transcripts generated during the original program evaluation. Researcher S.C.D., who holds a graduate certificate in qualitative research and inquiry methods, trained researcher M.G.H. in Braun and Clarke’s reflexive thematic analysis (RTA) [40,41], and the researchers collaborated throughout the analytic process.

Following Braun and Clarke’s RTA, the analytic process proceeded through iterative and recursive phases rather than a linear sequence. Consistent with RTA methodology, we first engaged in inductive familiarization with the data guided by “unmotivated looking,” remaining open to how data might suggest meaningful patterns rather than imposing a fixed framework from the outset [40,41,42]. Both researchers read each transcript multiple times and recorded initial impressions and analytic reflections in the memos.

During the coding phase, we generated initial descriptive codes that captured salient features of participants’ accounts. Coding was conducted inductively using MAXQDA (version 24.8) with ongoing analytic dialogue supporting reflexive engagement with the data rather than the pursuit of coding consensus. Codes were then examined and grouped into higher-order categories to construct preliminary themes representing shared patterns of meaning related to the mechanisms through which the CAM program supported mental well-being. In subsequent phases, themes were reviewed, refined, and reworked in relation to the coded data and the dataset as a whole. Through iterative discussion and analytic writing, themes were clarified, defined, and named to ensure internal coherence and conceptual distinctiveness. These reflexive processes culminated in the final set of themes presented in the Findings section. Theoretical frameworks were introduced only after themes were generated to support interpretation, rather than to structure or constrain the analytic process.

### 2.9. Reflexivity/Validity

This study was conducted within an interpretive qualitative framework, recognizing that both participants’ perspectives and our own perspectives as researchers inevitably shaped the analysis. Participants’ narratives reflected their individual interpretations of their lived experience, while our analytic lens was influenced by our prior knowledge, assumptions, and disciplinary training. In particular, our background in gerontology required attentiveness to ensure that we did not overemphasize themes aligned with our professional viewpoints unless the data supported such emphasis.

To strengthen reflexivity, we engaged in memo writing throughout the process and held regular team discussions to surface and question our assumptions. These practices helped us identify variability in accounts, give attention to perspectives voiced by a small number of participants, and deliberately highlight cases that challenged or contradicted dominant patterns voiced by most participants. We also enhanced validity through transparent documentation of coding decisions and the iterative development of a codebook that reflected both convergent and divergent perspectives.

## 3. Findings

The study included 10 older adults who had participated in the CAM program. Table 1 summarizes participant characteristics to provide contextual information about the sample. These descriptive data are presented to orient the reader and are not interpreted as analytic findings.

At the start of the CAM program, many older adult participants described internalized ageist beliefs that limited what they expected from their bodies and their lives. As one participant put it, “We’re old-fashioned, right? We’re little old ladies, we’re from the old days, right?” (P9); and another commented, “No, you can’t do everything, especially when you get older… it’s not going to happen.” (P2) These narratives provide the analytic context for the findings that follow.

Through participation in CAM, these assumptions were challenged as participants tried new behaviors, experienced small successes, and began to view themselves as capable of change. Notably, participants described physical progress—moving more, feeling stronger, managing pain—as connected to their emotional state. Improvements in physical capacity increased motivation and confidence. Thus, changes in mental well-being did not emerge separately from physical health, but through embodied experiences of capability and control. The findings that follow identify four themes that describe the mechanisms through which these shifts unfolded. A summary of these themes can be found in Table 2.

### 3.1. Belonging and Inclusion Through Trust and Safety

Many participants described feeling hesitant or unsure of the value of the CAM program before attending it for the first time. Some worried about being judged, about sharing personal information, or about whether they would be able to participate physically. These comments revealed a degree of internalized ageism that initially created barriers to their participation. In addition, some were socially isolated when they first began the CAM program, which may have exacerbated these feelings. However, these initial reservations quickly dissipated as participants engaged in the group health coaching sessions and began to feel emotionally safe, welcome, and included. One participant described the transition simply as: “I didn’t want to do this… But it was infectious.” (P2).

Belonging emerged from what participants described as a warm, non-judgmental environment—what one called “a safe space to just share what’s been going on.” (P6) Participants emphasized that health coaches and peer leaders created an atmosphere where they could relax, be themselves, and not feel self-conscious about their abilities or health challenges. As one participant shared, “I don’t feel judged. They just really put you at ease, and they know how to handle people who aren’t so much at ease in the beginning.” (P4) Another added, “Someone asks, ‘How do you feel?’ It’s like, okay, someone’s caring about how I feel today.” (P1).

The experience of feeling included was not only felt on an emotional level but also on a practical level: it was reinforced by the design and delivery of the CAM program. Participants consistently noted that the program was accessible regardless of age, physical ability, or confidence level: “Even if you’re heavy, if you’re old, if you’re homebound, you can still do it.” (P2) For others, belonging was reinforced by having the program delivered in their preferred language. One participant explained that when others chose to speak Spanish with her, “that’s how my friendships continued.” (P8).

As belonging deepened, participants realized they were not alone in their struggles. They found comfort and relief in seeing that others shared similar challenges: “You kind of get a feeling that you’re not the only one…we understood where each other was coming from because a lot of us had similar situations with our health and mobility.” (P6) For some, simply having a place to connect eased feelings of anxiety: “Sometimes I get nervous…but once I’m in the program, I feel calm.” (P10).

Through these experiences of being welcomed and included, participants described feeling part of something meaningful: “They’re definitely my family.” (P3) Belonging and inclusion laid the foundation for further engagement in the program, and participants said this made them more willing to show up, participate, and try new behaviors.

### 3.2. Personal Accountability Through Relational Accountability

Participants discussed the importance of personal accountability and described how that accountability emerged through relationships, e.g., being seen, being asked, and being expected back the next week. Saying goals aloud, being “witnessed” by others, and receiving gentle follow-up created a sense of responsibility that felt motivating rather than pressured. As one participant explained:

“When I’m sharing my [health] journey…for some reason I’m a little more accountable. You’re encouraged to say things out loud: ‘I want to do this.’ And you might get, ‘Well, how would you go about doing that?’”

(P4).

Rather than focusing on whether people followed a prescribed plan, participants said they were encouraged to be accountable to themselves. As P4 noted, “[They don’t ask] ‘did you eat your greens this weekend?’ They encourage you to be your best and be accountable to yourself.”

Accountability was supported not only through encouragement, but through practical structures that reduced barriers to action. Across participants, accountability became easier because they knew someone would notice if they were absent, struggling, or making progress. The consistency of being checked on strengthened follow-through: “Checking in on me… the next week, checking in on me, was critical for me to keep trying.” (P2).

Participants described accountability not as something negative but more as something they found helpful. Being asked how things were going and knowing others were also working to improve their health through making changes in health-related behaviors made participants feel capable of following through themselves. One participant noted that the program “creates a consciousness for taking care of oneself to have a better quality of life.” (P5).

In sum, these comments illustrate that accountability was not imposed; it was co-created through relationship, consistency, and the removal of practical barriers to change.

### 3.3. Self-Efficacy Through Social Learning and Reciprocal Support

Many participants initially voiced internalized beliefs that they could not make changes to improve their well-being. “I don’t walk much…because…it’s not the same like when one was young…and now as an old person…now the steps are very slow.” (P3). As participants gained a sense of belonging and accountability—both to the group and themselves—they observed other participants engaging in positive behaviors and began to see that this might be possible for themselves. While participants were generally positive about the intergenerational nature of the CAM program, they seemed most motivated by observing people like them (i.e., other older adults) and/or those who had similar chronic health challenges, take actions to improve their health. Recognizing that others were navigating similar health challenges helped participants feel less alone in their experience: “It showed me that I’m not the only one who is sick; there are many of us, and I see that they are moving forward. And that helps me.” (P10).

Seeing peers take action, especially while managing their own limitations, was deeply motivating. One participant described watching others take part in the movement portion of class while adapting to their abilities, such as holding onto a walker or completing movements from bed: “That really encouraged me. You think, oh, my knee hurts today. Well, there are other people that can’t get out of bed.” (P4).

Participants emphasized that while health coaches played an important role, the support they received from one another was deeply meaningful. While health coaches guided the structure of the program, learning from peers felt uniquely powerful: “Through the experiences shared by our peers, we learn even more.” (P5).

Further, participants described a sense of mutual encouragement in which support flowed in both directions and where giving support was as important as receiving it. This extended outside of the group coaching sessions; in addition to the health coaches, some participants checked in on each other during the week via supportive text messages. One participant described these mid-week messages as “reinforcing the feeling that we’re still here.” (P7).

This reciprocal support was described as motivating, energizing, and central to the group experience. Participants not only received help but also actively contributed to others’ progress. For many, supporting others became a key part of their own experience in the program. Participants said that offering encouragement—and seeing that it was appreciated—was meaningful: “It helped me to see that there are people that I can help. That’s another thing. I feel needed. I needed to feel needed.” (P2).

Through observing others, receiving encouragement, and encouraging others, participants built a foundation of self-efficacy, a belief that they could make changes to improve their own health and well-being.

### 3.4. Agency Through Positive Actions

Participants described gaining a sense of agency not simply from talking about change or believing it was possible, but from engaging in new behaviors and experiencing small successes. As they practiced habits such as eating differently, moving more, or practicing mindfulness, they began to observe progress, and these moments reinforced the belief that change was possible even though earlier in the program some participants felt limited by “being an old person now.” (P8).

For some, progress was physical and measurable:

“Now I can put this arm up. I can’t get the other one all the way up yet, but I can get this one up. It took a year–but hey.”

(P2).

Others noticed that new habits carried over into their daily routines:

“I check labels now… Before, I would just eat whatever. But now, I think about it.”

(P5).

Participants also described building routines outside the sessions. Movement, stress management, and healthier eating became part of daily life rather than something practiced only during the program. In addition, participants described improvements that extended beyond physical or behavioral changes. As routines became established and small successes accumulated, several participants reported an unexpected positive shift in how they felt day-to-day. “So long as you notice that there have been changes, that should motivate you to continue.” (P7).

One participant explained that the changes in her habits (e.g., eating differently, managing stress, sleeping better) coincided with improvements in her overall outlook:

“When I’m eating better, of course, I feel better. But I would say also it’s just my mental state. This program came to me at a very, very dark time in my life. The pandemic forced retirement, I lost my good medical insurance. There was a lot of scary things happening to me at the time, and I learned how to, number one, manage the stress better… I’m sleeping better… I just have overall better feelings about things. I don’t worry as much.”

(P4).

For this participant, engaging in small daily actions—such as preparing nutritious meals, trying new movement routines, or practicing mindfulness—helped her create a sense of stability and improve her outlook on life. Others echoed similar experiences, describing that, as they followed through on their goals and began to see progress, they felt more hopeful about the future. These emotional shifts emerged not from any single strategy but from the accumulation of small successes that participants practiced inside and outside the group.

As these small wins built on one another, participants began not only to feel better, but to act differently. They described feeling increasingly confident in acting on ideas they previously hesitated to try, which “helped me be brave enough to implement the ideas I have. I’m doing them now.” (P2). Several participants acknowledged seeing that shift in others, noting that: “People who came in with low self-esteem left very enthusiastic after taking the course.” (P7).

These experiences helped participants move from tentative experimentation to a sustained belief in their own capacity, reinforcing a sense of control and optimism that supported improved mental well-being.

### 3.5. Summary

**Figure 2:**
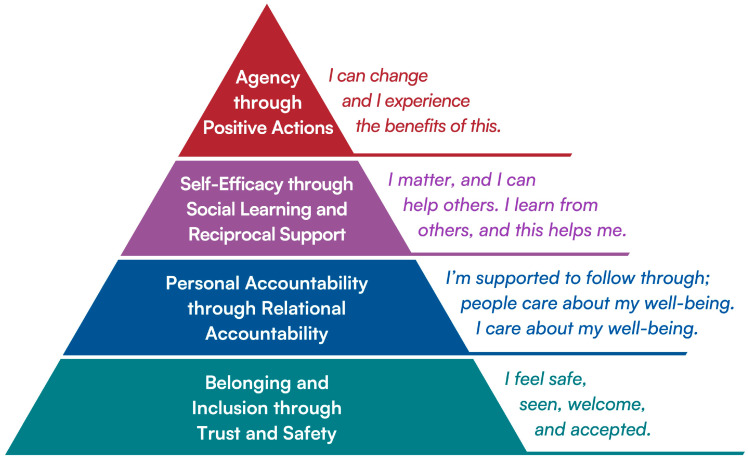
Conceptual model illustrating interrelated mechanisms described by participants.

Together, these themes point to a set of interconnected mechanisms through which participation in the CAM program supported mental well-being. Figure 2 illustrates how we conceptualized these relationships. We chose a pyramid intentionally, not to suggest a linear or stepwise progression, but to convey that these mechanisms function together rather than in sequence, and that no single element is sufficient on its own. Our analysis suggests that experiences of belonging, inclusion, trust, and safety serve as the foundation, creating the conditions that allow other mechanisms to take hold and endure over time.

## 4. Discussion

This study identified four themes representing interrelated mechanisms through which older adults described experiencing improved mental well-being while participating in the CAM program: (1) belonging and inclusion built through trust and safety; (2) personal accountability shaped through relational accountability; (3) self-efficacy supported through social learning and reciprocal support; and (4) agency reinforced through positive actions. These mechanisms were not experienced as discrete or strictly sequential but as overlapping and mutually reinforcing aspects of the group-based health coaching environment. Rather than any single factor being cited as most important or impactful, participants described a relational and experiential non-linear process in which feeling included, supported, capable, and able to act meaningfully co-occurred and deepened over time.

Consistent with this interpretive pattern, research suggests that improvements in mental well-being among older adults often emerge through an interplay of behavioral engagement, emotional experience, and psychological resources. Physical activity, for example, is associated with a higher quality of life not solely because it improves physical function, but because it supports mood, confidence, social engagement, and daily functioning [43]. Similarly, positive emotions experienced through practicing health-supportive behaviors have also been shown to contribute to “upward spirals” that strengthen motivation and build durable psychological resources such as confidence, resilience, and social connection [44]. Self-efficacy also consistently predicts health behaviors and overall health status among older adults and may help buffer the effects of socioeconomic disadvantage [45]. Together, this body of evidence provides context for how participants in the CAM program described small, meaningful behavioral successes as emotionally significant and motivating, and how these experiences supported improved mental well-being.

Although our analysis was not driven by a predetermined framework, our findings align with the Social Ecological Model [33,46]. Belonging reflects interpersonal and community-level influences; relational accountability reflects interpersonal expectations and shared norms reinforced by the program’s structure; reciprocal support—including both learning through observation and through giving and receiving encouragement—reflects gains in self-efficacy through individual, interpersonal, and community-level actions; and agency reflects individual-level psychological change, including increased confidence and ability [33,46]. CAM, therefore, operates across ecological levels rather than placing responsibility solely on individual behavior; it fosters social environments and experiential learning opportunities that support meaningful behavioral change. This broader, multilevel influence is consistent with mental well-being research showing that emotional health in later life is shaped not only by social contact, but by environments that foster identity, psychological safety, and meaning, and opportunities to contribute to and learn from others [15,16,17,20,47].

In this study, belonging and inclusion through trust and safety emerged as a central relational mechanism supporting mental well-being. Rather than focusing solely on individual experiences, this theme highlights how identity-safe, nonjudgmental group environments create conditions in which older adults can engage without fear of exclusion or inadequacy [48]. Research suggests that such inclusive social environments are particularly important for older adults experiencing social loss or marginalization, as they help preserve emotional well-being and reduce psychological distress [48]. When trust and safety are established, belonging can function as a stabilizing psychosocial resource that supports ongoing engagement and emotional regulation, rather than merely increasing social contact [20,48]. Consistent with prior research, older adults who feel they belong are more motivated to engage socially, and such engagement becomes a source of positive feelings, meaning, and fulfillment [48]. In this way, belonging operates as a protective mechanism, buffering the harmful emotional effects of social disconnection; research shows a sense of belonging lowers depressive symptoms and feelings of hopelessness [49].

Personal accountability through relational accountability functioned as a key interpersonal mechanism supporting mental well-being. Rather than treating accountability as externally imposed or compliance-based, this mechanism reflects how accountability can be embedded within supportive relationships and shared expectations. Relational accountability operates through being witnessed, encouraged, and gently followed up with, creating a context in which individuals feel both supported and responsible to themselves and to others [50]. This interpersonal process aligns with theoretical work describing accountability as a “relational virtue” that is responsive to others and grounded in shared commitments, and that contributes to healthy autonomy, self-regulation, and human flourishing [51].

Self-efficacy through social learning and reciprocal support was identified as a key psychosocial mechanism supporting mental well-being. Self-efficacy is particularly relevant in this context, as it has been shown to mediate the effects of loneliness, anxiety, depression, and physical health challenges among older adults, including those who are marginalized or living with multimorbidity [52,53,54]. Rather than relying on direct instruction or persuasion, this mechanism reflects how confidence in one’s own capability can be supported through social learning, e.g., learning from watching others try new behaviors, adapting them to their own circumstances, and continuing despite challenges. Social cognitive theory identifies observational learning as a foundational process through which self-efficacy develops, particularly when individuals observe others facing similar limitations or health challenges [55,56]. In this way, social learning helps normalize their effort and makes change feel attainable rather than exceptional.

Reciprocal support further strengthens the self-efficacy mechanism by positioning individuals not only as recipients of encouragement but also as contributors to others’ progress. While social support is widely recognized as beneficial for physical and mental health, support that flows in both directions highlights the active dimensions of connection—giving as well as receiving—that reinforce competence, purpose, and social identity [52,57,58,59]. Prior research shows that providing support is associated with reduced stress reactivity, lower depressive symptoms, and even reduced mortality risk, and that contributing to others fosters purpose and belonging in later life [16,17,60].

Agency through positive actions functioned as a key psychological mechanism supporting mental well-being. Rather than conceptualizing agency as a stable trait, this mechanism reflects how opportunities to take action and experience small, achievable successes can support a sense of personal control and capability over time [55,61]. Prior research demonstrates that enhancing perceptions of personal control is associated with significant benefits for older adults, including improved physical, cognitive, and psychological health, as well as better pain management [62,63,64]. While some population studies find that perceived control declines after midlife [65,66], others argue that this erosion may reflect internalized ageism rather than inevitable age-related decline [14]. Because agency reflects a person’s belief in their ability to influence their own life, these gains have important implications for mental well-being, as higher levels of agency are associated with lower depression, lower anxiety, and greater life satisfaction [62,67]. Within group-based health promotion contexts, agency can be supported through repeated experiences of action and feedback that reinforce a sense of personal capability, particularly in environments that counter age-related stereotypes and support experimentation without fear of failure [14,55,63].

Overall, these findings reveal that the CAM program is experienced as supporting core psychological capacities relevant to mental well-being, rather than solely increasing social connection. This aligns with the World Health Organization’s definition of mental health as the ability to cope with everyday challenges, recognize one’s abilities, and contribute meaningfully to one’s community [68]. Participants’ accounts suggest that the CAM program supported ways of seeing themselves as capable contributors to their own well-being.

### Strengths and Limitations

A key strength of this study is the depth and richness of the qualitative data, which allowed us to examine not only what changed for participants but also how those changes unfolded over time—that is, the factors that motivated and supported them. Using a secondary analysis approach also enabled us to revisit the data through fresh conceptual lenses, yielding insights not visible in the primary evaluation. In addition, participants were diverse in terms of race, gender, preferred language, background, and health issues. This offered perspectives often missing from research on mental well-being in later life.

This study has limitations. The sample was drawn from a single community-based program, and participants who agreed to interviews may represent individuals who were more engaged or who experienced more positive outcomes. Because this was a secondary analysis, we were limited to the questions and prompts used in the original interviews. Future research that intentionally examines the factors supporting improved mental well-being among older adults participating in the CAM program could deepen and extend these findings.

## 5. Conclusions

This study suggests that improving mental well-being in later life requires more than increasing social contact; it requires relational environments that support participation, shared practice, and growing confidence in one’s ability to make meaningful changes. In Open Source Wellness’s CAM program, trust and safety, relational accountability, social learning, and reciprocal support enabled older adults to try new behaviors, witness one another’s progress, and participate in ways that fostered confidence, meaning, and agency. These relational and experiential mechanisms helped participants revise internalized beliefs about aging and possibility and supported engagement in behaviors that enhanced mental well-being. Programs that aim to support healthy aging may benefit from designing environments that enable shared practice and meaningful contribution, particularly for older adults in under-resourced communities who have fewer opportunities for supportive, identity-affirming engagement. Taken together, these findings suggest that efforts to support mental well-being in later life should move beyond simply creating opportunities for social interaction and instead prioritize relational experiences that help older adults see themselves as capable, valued, and able to influence their own health and lives. Future research should examine if and how relational and experiential elements of group-based interventions contribute to sustained behavior change over time, and whether these can be adapted across diverse community, cultural, and healthcare settings.

## Figures and Tables

**Table 1:** Participant characteristics (*n* = 10).

Participant # *	Age When Interviewed	Gender	Preferred Language
1	68	F	English
2	70	F	English
3	66	M	English
4	66	F	English
5	72	M	Spanish
6	65	F	English
7	66	F	Spanish
8	84	F	Spanish
9	65	F	Spanish
10	73	F	Spanish

* Each de-identified transcript was assigned a number for identification and tracking purposes.

**Table 2:** Themes.

Theme	Definition	Representative Quotes
Belonging and Inclusion through Trust and Safety	Belonging emerged from emotional safety, kindness, and non-judgment from both peers and coaches, as well as intentional program design choices that made participation accessible regardless of age, mobility, language, or confidence level.	“I think the main thing that I find most inviting is the non-intimidation of everything…And I really like that.” (P4)
Personal Accountability through Relational Accountability	Personal accountability emerged from participants taking responsibility for their choices and for engaging with the program. This accountability was strengthened through relationships. Sharing goals aloud, being witnessed by others, and receiving gentle follow-up from coaches and peers made follow-through more achievable.	“And that’s one of the good things that comes back…the goal and also the accountability to reach your goals.” (P3)
Self-Efficacy through Social Learning and Reciprocal Support	Self-efficacy came from seeing peers try new behaviors, which served as powerful role modeling. It was strengthened from being supported by other group members and from contributing to the group and uplifting others.	“You learn from the people there and they also learn from you.” (P5)
Agency Through Positive Actions	Agency came from trying new behaviors and experiencing small successes. As participants practiced new habits such as eating differently, moving more, setting boundaries, or trying new activities, they saw tangible progress in their bodies, routines, or confidence. These “I did it” moments generated momentum: success increased their belief in their own capability and helped them internalize a new identity: “I am someone who can change.”	“If you’re willing to put the effort in, you get a lot out of it. Well, in my case, I lost about 10, 15 pounds.” (P3)

## Data Availability

Upon reasonable request, data are available from the corresponding author.

## Abbreviations

The following abbreviations are used in this manuscript

WHO	World Health Organization
CAM	Community As Medicine
SDOH	Social Determinants of Health
RTA	Reflexive Thematic Analysis
R4H	Recipe4Health (R4H)
FQHC	Federally qualified health center

## References

[1] Joshi M., Finney N., Hale J.M. (2024). Loneliness and social isolation of ethnic minority/immigrant older adults: A scoping review. Ageing Soc..

[2] Office of the Surgeon General (OSG) (2023). Our Epidemic of Loneliness and Isolation: The U.S. Surgeon General’s Advisory on the Healing Effects of Social Connection and Community.

[3] World Health Organization (2025). From Loneliness to Social Connection: Charting a Path to Healthier Societies: Report of the WHO Commission on Social Connection.

[4] Miyawaki C.E. (2015). Association of social isolation and health across different racial and ethnic groups of older Americans. Ageing Soc..

[5] Malcolm M., Frost H., Cowie J. (2019). Loneliness and social isolation causal association with health-related lifestyle risk in older adults: A systematic review and meta-analysis protocol. Syst. Rev..

[6] Holt-Lunstad J., Hudson R.B. (2018). The Potential Public Health Relevance of Social Isolation and Loneliness. Public Policy Aging Rep..

[7] Cacioppo J.T., Hughes M.E., Waite L.J., Hawkley L.C., Thisted R.A. (2006). Loneliness as a specific risk factor for depressive symptoms. Psychol. Aging.

[8] Ellwardt L., Aartsen M., Deeg D., Steverink N. (2013). Does loneliness mediate the relation between social support and cognitive functioning?. Soc. Sci. Med..

[9] Jakobsson U., Hallberg I.R. (2005). Loneliness, fear, and quality of life among elderly in Sweden: A gender perspective. Aging Clin. Exp. Res..

[10] Newman-Norlund R.D., Newman-Norlund S.E., Sayers S., Mclain A.C., Riccardi N., Fridriksson J. (2022). Effects of social isolation on quality of life in elderly adults. PLoS ONE.

[11] Naito R., Mckee M., Leong D., Bangdiwala S., Rangarajan S., Islam S., Yusuf S. (2023). Social isolation as a risk factor for all-cause mortality. PLoS ONE.

[12] Allen J.O. (2015). Ageism as a risk factor for chronic disease. Gerontologist.

[13] Kang H., Kim H. (2022). Ageism and psychological well-being among older adults. Gerontol. Geriatr. Med..

[14] Levy B. (2009). Stereotype embodiment: A psychosocial approach to aging. Curr. Dir. Psychol. Sci..

[15] Bar-Tur L. (2021). Fostering well-being in the elderly. Front. Med..

[16] Inagaki T.K., Eisenberger N.I. (2016). Giving support to others reduces stress responses. Psychophysiology.

[17] Jetten J., Haslam S.A., Cruwys T., Greenaway K.H., Haslam C., Steffens N.K. (2017). Advancing the social identity approach to health. Eur. J. Soc. Psychol..

[18] Applewhite A. (2016). This Chair Rocks: A Manifesto Against Ageism.

[19] Sonke J., Manhas N., Belden C., Morgan-Daniel J., Akram S., Marjani S., Fancourt D. (2023). Social prescribing outcomes. Front. Med..

[20] Flett G.L. (2015). An introduction and conceptual analysis of mattering. J. Psychoeduc. Assess..

[21] US Department of Health and Human Services Healthy People 2030. https://health.gov/healthypeople.

[22] Grenier A., Phillipson C., Laliberte Rudman D., Hatzifilalithis S., Kobayashi K., Marier P. (2017). Precarity in late life. J. Aging Stud..

[23] Singer L., Green M., Rowe F., Ben-Shlomo Y., Morrissey K. (2019). Social determinants of multimorbidity. SSM Popul. Health.

[24] Byun M., Kim E., Ahn H. (2021). Factors contributing to poor self-rated health. Healthcare.

[25] Byers A.L., Arean P.A., Yaffe K. (2013). Low Use of Mental Health Services. Psychiatr. Serv..

[26] Choi N.G., Kunik M.E., Wilson N. (2013). Mental health service use among low-income adults. J. Aging Health.

[27] Elshaikh U., Sheik R., Saeed R.K.M., Chivese T., Hassan D.A. (2023). Barriers to mental health help-seeking. BMC Geriatr..

[28] Reynolds C.F., Jeste D.V., Sachdev P.S., Blazer D.G. (2022). Mental health care for older adults. World Psychiatry.

[29] Emmert-Aronson B., Grill K.B., Trivedi Z., Markle E.A., Chen S. (2019). Group medical visits 2.0. J. Altern. Complement. Med..

[30] Duplantier S.C., Lee J., Markle E.A., Emmert-Aronson B. (2025). Community as Medicine: A Novel Approach to Improve Health Behaviors and Mental Well-Being for Vulnerable Populations. Am. J. Lifestyle Med..

[31] American College of Lifestyle Medicine Redefining Healthcare. https://lifestylemedicine.org.

[32] Bronfenbrenner U. (1977). Toward an experimental ecology of human development. Am. Psychol..

[33] Mcleroy K.R., Bibeau D., Steckler A., Glanz K. (1988). An ecological perspective on health promotion programs. Health Educ. Q..

[34] Thompson-Lastad A. (2019). Group medical visits as participatory care. Qual. Health Res..

[35] Heaton J. (2008). Secondary analysis of qualitative data: An overview. Historical Social Research..

[36] Hinds P.S., Vogel R.J., Clarke-Steffen L. (1997). Secondary analysis of qualitative data. Qual. Health Res..

[37] Sherif V. (2018). Evaluating preexisting qualitative research data. Qual. Soc. Work.

[38] Van Nes F., Abma T., Jonsson H., Deeg D. (2010). Language differences in qualitative research. Eur. J. Ageing.

[39] Squires A. (2010). Methodological challenges in cross-language research. Int. J. Nurs. Stud..

[40] Braun V., Clarke V. (2006). Using thematic analysis in psychology. Qual. Res. Psychol..

[41] Braun V., Clarke V. (2020). One size fits all?. Qual. Res. Psychol..

[42] O’Reily M., Lester J. (2019). Applied conversation analysis. Couns. Psychother. Res..

[43] McAuley E., Szabo A., Gothe N., Olson E.A. (2011). Self-Efficacy and physical activity. Am. J. Lifestyle Med..

[44] Van Cappellen P., Rice E.L., Catalino L.I., Fredrickson B.L. (2018). Positive affective processes. Psychol. Health.

[45] Grembowski D., Patrick D.L., Diehr P., Durham M., Beresford S., Kay E., Hecht J. (1993). Self-efficacy and health behavior. J. Health Soc. Behav..

[46] Bronfenbrenner U. (1979). The Ecology of Human Development.

[47] Saadeh M., Welmer A.K., Dekhtyar S., Fratiglioni L., Calderón-Larrañaga A. (2020). Psychological well-being and physical function. J. Gerontol. A Biol. Sci. Med. Sci..

[48] Portacolone E., Johnson J.K., Halpern J., Kotwal A. (2023). Seeking a Sense of Belonging. Gerontologist.

[49] Fisher L.B., Overholser J.C., Ridley J., Braden A., Rosoff C. (2015). Sense of belonging and suicide risk. Psychiatry.

[50] Thoits P.A. (2011). Mechanisms linking social ties to health. J. Health Soc. Behav..

[51] Peteet J.R., Witvliet C.V.O., Evans C.S. (2022). Accountability and mental health. Philos. Psychiatry Psychol..

[52] Paukert A.L., Pettit J.W., Kunik M.E., Wilson N., Novy D.M., Rhoades H.M., Greisinger A.J., Wehmanen O.A., Stanley M.A. (2011). Social support and self-efficacy. J. Clin. Psychol. Med. Settings.

[53] Roskoschinski A., Liang W., Duan Y., Al-Salehi H., Lippke S. (2023). Loneliness and depression. Front. Psychiatry.

[54] Kwon S., Benoit E., Windsor L. (2024). Social support during COVID-19. BMC Geriatr..

[55] Bandura A. (1977). Self-efficacy: Toward a unifying theory. Psychol. Rev..

[56] Fryling M.J. (2019). Understanding Observational Learning.

[57] Holt-Lunstad J. (2022). Social connection as a public health issue. Annu. Rev. Public Health.

[58] Harvey I.S., Alexander K. (2013). Perceived social support. J. Cross Cult. Gerontol..

[59] Ozbay F., Fitterling H., Charney D., Southwick S. (2007). Social support and resilience. Curr. Psychiatry Rep..

[60] Brown S.L., Nesse R.M., Vinokur A.D., Smith D.M. (2003). Providing social support. Psychol. Sci..

[61] Robinson S.A., Lachman M.E. (2017). Perceived control and aging. Gerontology.

[62] Mallers M.H., Claver M., Lares L.A. (2013). Perceived control in older adults. Gerontologist.

[63] Langer E.J., Rodin J. (1976). Effects of choice for the aged. J. Personal. Soc. Psychol..

[64] Rodin J., Langer E.J. (1977). Long-term effects of control interventions. J. Personal. Soc. Psychol..

[65] Moore J.W. (2016). Sense of agency. Front. Psychol..

[66] Drewelies J., Wagner J., Tesch-Römer C., Heckhausen J., Gerstorf D. (2017). Perceived control across life. Psychol. Aging.

[67] Corrigan J.A., Schutte N.S. (2023). Hope dimensions and depression. Int. J. Appl. Posit. Psychol..

[68] World Health Organization Mental Health: Strengthening Our Response. Updated 8 October 2025. https://www.who.int/news-room/fact-sheets/detail/mental-health-strengthening-our-response.

